# The Use of Tenofovir Disoproxil Fumarate in the Management of eAg-Negative Chronic Hepatitis B Infection

**DOI:** 10.3390/jcm13071864

**Published:** 2024-03-24

**Authors:** Nishita Jagarlamudi, Melissa Reyes, Scott Fung, Florence Wong

**Affiliations:** Division of Gastroenterology and Hepatology, Toronto General Hospital, University Health Network, University of Toronto, Toronto, ON M5G2C4, Canadamelissa.reyes@uhn.ca (M.R.); scott.fung@uhn.ca (S.F.)

**Keywords:** chronic hepatitis B, tenofovir, inactive carrier, functional cure

## Abstract

**Background/Objectives:** Currently, there are insufficient data to recommend the treatment of patients with hepatitis B e antigen (HBeAg)-negative chronic infection who have normal ALT and low HBV DNA, since the prognosis is generally regarded as favorable. The aim of this pilot study was to determine whether the use of tenofovir disoproxil fumarate (TDF) 300 mg/day for 3 years was able to achieve functional cure (HBsAg loss) and HBsAg seroconversion in HBeAb-positive individuals. **Methods:** Fifty patients not on antiviral therapy (40% men, mean age 48.9 ± 10.9 years, 84% Asians) with minimal fibrosis were enrolled. **Results:** TDF reduced HBV DNA significantly to undetectable levels after 6 months. Overall, 48.3% of inactive carriers (baseline HBV DNA < 2000 IU/mL) remained HBV DNA negative 6 months after treatment withdrawal, which was significantly higher than the 5.6% in those who were not inactive carriers (baseline HBV DNA ≥ 2000 IU/mL) (*p* = 0.003). The HBsAg levels did not drop throughout the study period with no difference between inactive carriers versus those who were not. Five inactive carriers achieved functional cure, but none of these were amongst those who were not inactive carriers. No renal dysfunction or ALT flare on treatment withdrawal was observed. **Conclusions:** TDF could potentially be used to induce functional cure in patients who are inactive carriers with normal ALT, low HBV DNA and without advanced fibrosis.

## 1. Introduction

Chronic hepatitis B (CHB) viral infection is a major global health issue. In 2019, the World Health Organization estimated that 296 million people were positive for hepatitis B surface antigen (HBsAg), and the global prevalence is approximately 3.5% [1]. The prevalence of CHB infection varies widely in different parts of the world: it is highest in East Asia and sub-Saharan Africa and lowest in North America and Western Europe. Canada is a country with a long history of immigration; in 2021, 23% of the population in Canada were immigrants [2], and many were born in hepatitis B virus (HBV) endemic countries. Currently, it is estimated that there are approximately 250,000–460,000 people in Canada who are living with CHB infection [3].

The natural history of CHB infection is quite complex but can be divided into several phases depending on the level of viral replication. Most adults who were infected during infancy have gone through a phase of immune-mediated viral clearance and entered a phase of relative inactivity with respect to viral replication and liver inflammation [4]. Such patients typically have repeatedly normal alanine aminotransferase (ALT) levels, HBV deoxyribonucleic acid (DNA) of <2000 IU/mL and are HBsAg positive, but they are also hepatitis B e antigen (HBeAg) negative, and hepatitis B e antibody (HBeAb) positive. Liver biopsy typically shows minimal or no inflammatory activities. The course of most of these patients is usually benign, and therefore, current treatment guidelines [5,6,7] recommend regular monitoring rather than the treatment of these patients. However, recent evidence suggests that the prognosis of patients with inactive CHB is not entirely benign. Rarely, patients, even non-cirrhotic, may develop hepatocellular carcinoma (HCC) despite inactive infection [8]. In addition, approximately 20 to 30% of patients with inactive CHB may undergo a spontaneous reactivation of hepatitis B during follow-up [9]. Because these patients do not uniformly have a benign course, the term ‘inactive carrier’ is being replaced by the term ‘HBeAg-negative chronic infection’. Furthermore, there is significant stigma associated with CHB infection [10,11]; therefore, many patients have a strong desire to eliminate CHB despite not meeting criteria for antiviral therapy by current guidelines. However, it remains unclear whether the treatment of patients with HBeAg-negative chronic infection can reduce the risk of adverse liver outcomes [12]. Therefore, the aim of this study was to assess whether tenofovir disoproxil fumarate (TDF) can induce a loss of HBsAg in subjects with HBeAg-negative chronic infection.

## 2. Patients and Methods

This was an open-label, pilot study consisting of consecutive adult patients with HBeAg-negative chronic infection, which was defined as an absence of HBeAg, low HBV DNA levels < 4 log IU/mL and persistently normal or near-normal ALT for more than 6 months. Exclusion criteria included age over 75 years, co-infection with hepatitis C virus, hepatitis D virus, human immunodeficiency virus, abnormal renal function defined by a baseline serum creatinine of >110 µmol/L, a history of decompensated cirrhosis as defined by having had variceal bleed, ascites, or hepatic encephalopathy in the past, and HCC receiving treatment. 

Once patients fulfilled inclusion and exclusion criteria, baseline blood work including complete blood count, renal function, electrolytes, liver enzymes and liver function tests, as well as HBV serology including quantitative HBsAg, HBsAb, HBeAg, HBeAb and HBV DNA were collected at baseline. Patients also underwent transient elastography (Fibroscan) to document the stage of fibrosis and an abdominal ultrasound to exclude the presence of HCC. After the completion of all investigations, patients were started on TDF 300 mg (Gilead Sciences Inc., Foster City, CA, USA) once daily. Patients were reviewed at 6-monthly intervals. At each visit, patients received the same blood tests as above. A pill count was made by study staff to ensure medication compliance. Patients also had repeat abdominal ultrasound and Fibroscan on an annual basis. Treatment was discontinued with HBsAg loss or seroconversion to HBsAb or at the end of 3 years, whichever occurred first.

The primary endpoint was HBsAg loss; secondary endpoints were (i) clinical relapse defined as a rise in ALT ≥ 1.5 times the upper limit of normal accompanied by a rise in HBV DNA of ≥2 log IU/mL, (ii) progression of hepatic fibrosis by noninvasive testing and (iii) hepatic complications including HCC and decompensation.

The study was approved by the Research Ethics Board of the University Health Network and was registered at Clinicaltrial.gov (NCT02600117). The date of Ethics Committee approval was 22 May 2015. All authors had access to the data and approved the final manuscript.

## 3. Statistical Analysis

A descriptive analysis was performed for variables of the study for the baseline and all post-baseline measurements. Continuous variables were described using the means, medians, standard deviation, 95% confidence interval, minimum and maximum values of each distribution. Categorical variables were described using the frequencies and percentage of each category. Comparison between baseline and post-treatment period used Student’s *t*-test for paired data. For variables that did not have a normal distribution, the analysis of the changes from the baseline was performed using nonparametric tests (Wilcoxon signed-ranked test). For repeated measures, two-sided *p*-values were calculated using a repeated measures model with unstructured covariance with visit as a fixed effect. As this was a pilot study, no formal sample size calculation was made. All statistical tests were two-sided, using the threshold α < 0.05 to determine statistical significance. Analyses were performed in Prism 10 (Graphpad, Boston, MA, USA).

## 4. Results

Between January 2016 and July 2019, 61 patients were screened, and 50 patients were enrolled. The reasons for screen failures include HBV DNA either ≥4 log IU/mL (n = 2) or <10 IU/mL (n = 1), elevated ALT at screening (n = 5), withdrawal of consent (n = 2), and investigator’s decision to withdraw patient (n = 1) (Supplementary Figure). There were 20 men and 30 women with a mean age of 48.9 ± 10.9 years. Forty-two patients (84%) were Asian, 4 (8%) were of African descent and 4 (8%) were Caucasian. The mean duration of known HBV diagnosis was 15.2 ± 9.2 years. Thirty-seven (74%) patients were treatment naïve, while the remaining patients had received various antiviral treatment for a median of 9.5 months at a mean of 14 ± 4 years before enrollment. This included interferon (n = 3), lamivudine (n = 9), and unknown (n = 2). One patient had sequential prior treatment of 4 months of interferon followed by 36 months of lamivudine. Patients who received interferon had a shorter duration of prior treatment of 2 to 6 months, while those who received lamivudine had a median of 22 months of treatment. Prior interferon treatment was stopped because the duration of treatment was dictated by guidelines that were existing at that time. Lamivudine treatment was stopped because patients seroconverted from HBeAg positive to HBeAb positive. At the time the patients received their prior treatment, TDF was not yet available. Baseline and follow-up laboratory values are shown in Table 1. All patients underwent an abdominal ultrasound, which confirmed the absence of cirrhosis and HCC. The mean baseline Fibroscan score was 5.2 ± 1.6 kPa, and the mean baseline Fibrotest score was 0.23 ± 0.15, confirming the absence of significant hepatic fibrosis in these patients.

One patient withdrew from the study after one week, reporting nausea on TDF, another patient withdrew at 6 months as recommended by her nephrologist after diagnosis of polycystic kidney disease, and one patient withdrew at 18 months because of an unplanned pregnancy. One patient underwent HBsAg seroconversion at 18 months and completed the study early. The remaining 46 patients remained for the entire duration of the study. Table 1 shows serial laboratory results. All patients maintained normal liver enzymes throughout the study, and the differences between the various visits were statistically significant but not clinically significant (Table 1). Liver function parameters were also normal. There was no significant change in renal function in any of the patients, and the estimated glomerular filtration rate was stable and normal throughout the study (Table 1). The Fibroscan score remained stable and unchanged throughout the study period (Table 1). There were statistically significant differences noted in Fibrotest scores between the study visits, but the differences were clinically insignificant (Table 1).

**Figure 1:**
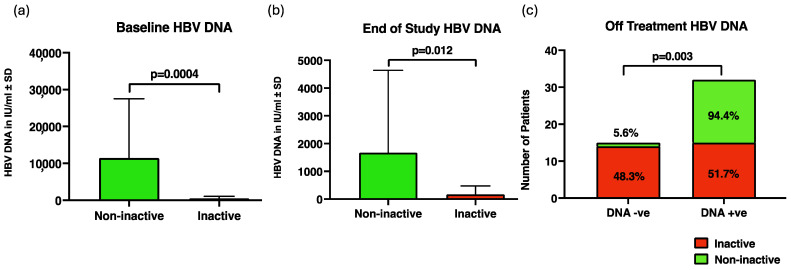
HBV DNA levels at (**a**) baseline and (**b**) at end of treatment; (**c**) the proportion of patients who remained HBV DNA negative 6 months after completion of treatment between those who were and who were not true inactive carriers.

Thirty-one of the fifty enrolled patients were inactive carriers (defined as HBeAg negative, low HBV DNA of <2000 IU/mL, and persistently normal or near-normal ALT) [13]. At baseline, inactive carriers had a median HBV DNA of 213 IU/mL (interquartile range or IQR: 65–827 IU/mL), which was significantly lower than that of those who were not inactive carriers (median: 5860 IU/mL; IQR: 4520–9100 IU/mL) (*p* = 0.004) (Figure 1a). Treatment with TDF resulted in a rapid decline of HBV DNA in both groups; from 12 months onwards, 46/48 patients had persistently negative HBV DNA (Table 1). At the end of the 3-year treatment period, all inactive carriers (n = 29) continued to have undetectable HBV DNA, while only two of those who were not inactive carriers (n = 18) showed undetectable HBV DNA. Six months after withdrawal of TDF treatment, the median HBV DNA in inactive carriers continued to be significantly lower than those who were not inactive carriers (9 IU/mL; IQR: 0–199 IU/m vs. 126 IU/mL; IQR: 68–2070 IU/mL) (*p* = 0.012) (Figure 1b). In addition, there were significantly more inactive carriers who remained HBV DNA negative (14/29 or 48.3%) compared to those who were not inactive carriers (1/18 or 5.6%) (*p* = 0.003) (Figure 1c).

**Figure 2:**
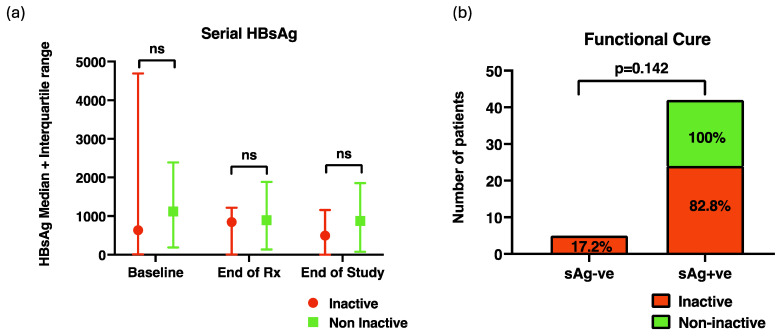
(**a**) Serial HBsAg levels at various time points during the study period between those who were and who were not true inactive carriers; (**b**) the proportion of patients who achieved functional cure 6 months after completion of treatment between those who were and who were not true inactive carriers. ns: not significant; Rx: treatment.

Quantitative HBsAg levels did not show any significant change during the study period. There was also no significant difference in HBsAg levels between inactive carriers and those who were not active carriers (Figure 2a). At 6 months after TDF withdrawal, there were five (16.1%) patients who achieved functional cure (undetectable HBsAg and HBV DNA 6 months after treatment withdrawal) amongst inactive carriers (Figure 2b), and four (13.8%) of these five patients achieved seroconversion. None of the patients who achieved a functional cure or seroconverted from HbsAg positive to HbsAb positive received prior antiviral therapy. Two of the four patients who seroconverted also had HBV DNA of <100 IU/mL at the commencement of treatment. We were unable to identify any clinical or laboratory features that could predict HbsAg seroconversion. No case of functional cure was observed amongst those who were not inactive carriers.

Liver enzymes remained within normal limits for all patients during treatment and the follow-up period. None of the patients developed renal dysfunction throughout the study period (Table 1). There were no ALT flares and no evidence of clinical or virologic relapse after TDF withdrawal, and no patient required re-treatment with TDF during the study. No patient developed cirrhosis or HCC as documented on annual screening abdominal ultrasound.

## 5. Discussion

HBeAg-negative chronic infection or the inactive carrier state is a distinct phase in the natural history of chronic hepatitis B infection characterized by normal ALT and low serum HBV DNA of <2000 IU/mL. It follows the immune clearance phase when hepatitis B inflammatory activity settles down. The prognosis of these patients is usually regarded as benign, especially if liver injury before HBeAg seroconversion is mild [14,15]. However, recent studies have shown that some patients with HBeAg-negative chronic infection are in fact ‘gray-zone’ patients who have ongoing HBV replication and HBV DNA levels fluctuate between 2000 and 20,000 IU/mL [16]. While truly inactive carriers have a very low incidence of progression to cirrhosis or develop HCC even after more than 10 years of follow-up [14], those with higher HBV DNA levels may have intermittent necro-inflammation and therefore may eventually develop advanced fibrosis during long-term follow-up [17]. Therefore, HBeAg-negative gray-zone patients are to be distinguished from truly inactive carriers who have lower HBsAg and HBV DNA levels, as the former group may benefit from antiviral treatment. Predictors of ALT flares amongst HBeAg-negative patients include male gender, higher baseline HBV DNA, and infection with genotype C [18,19].

**Figure 3:**
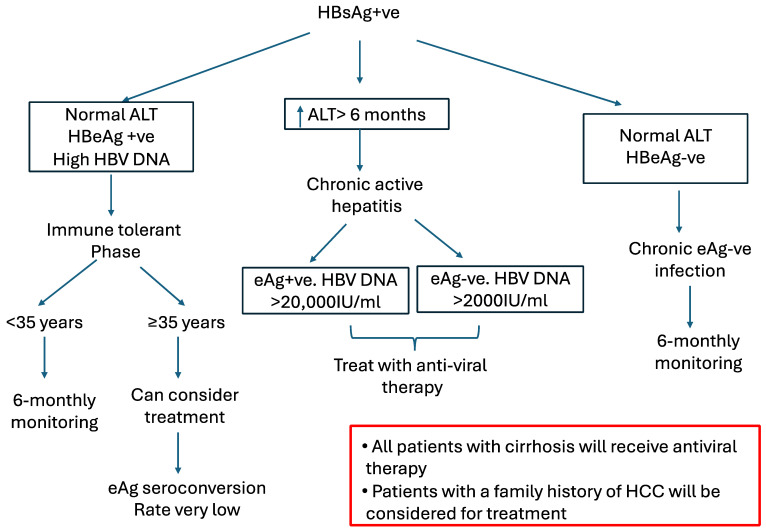
The local treatment algorithm for patients with chronic hepatitis B infection. ALT: alanine aminotransferase; DNA; deoxyribonucleic acid; HBV: hepatitis B virus; sAg: surface antigen; eAg: e antigen.

Treatment guidelines from Western countries suggest monitoring with ALT and HBV DNA every 6 to 12 months but not treatment for inactive carriers along with non-invasive testing for fibrosis every 1 to 2 years [6,7,12]. For those patients who are HBeAg negative but whose baseline HBV DNA is ≥2000 IU/mL, treatment is recommended if there is more than mild inflammation on liver biopsy or significant fibrosis on non-invasive testing [6,7,12,20]. The local treatment algorithm for patients with chronic hepatitis B is shown in Figure 3. However, recent treatment guidelines from China have recommended treating all HBsAg-positive patients who have a history of cirrhosis, liver failure liver transplantation, HCC or those who are about to be immune suppressed [21]. Those who are HBsAg positive with detectable DNA and ALT less than the upper limit of normal are treatment-eligible irrespective of HBeAg status or HBV DNA level if they have a family history of HCC or cirrhosis, are over 30 years, have advanced hepatic fibrosis or cirrhosis or have extrahepatic manifestations of chronic hepatitis B. This simplification in treatment guidelines is intended to expand treatment indications and increase the number of patients who receive antiviral therapy including those with HBeAg-negative chronic infection. The rationale for the expansion of treatment guidelines is to include patients with HBeAg-negative chronic infection or gray-zone patients who have intermittent flares in ALT and HBV DNA which may go undetected for lack of regular monitoring. This subset of patients may comprise a significant proportion of HBeAg-negative patients as indicated in one report, which showed that up to 50% of all HBeAg-negative patients were classified as gray-zone patients [22] who may have been overlooked by current treatment guidelines due to the inability to clearly classify such patients. Several studies have reported improved clinical outcomes in gray-zone patients who received long-term nucleoside analog therapy. In a study of over 450 patients in Taiwan, a subset of gray-zone patients was found to be at high risk for the development of HCC, leading the authors to conclude that they would have benefited from antiviral treatment [23]. Unfortunately, hepatitis B surface antigen loss or seroconversion was not reported. 

Similar to these reports, the majority of the patients in this study were classified as inactive carriers and therefore would receive monitoring without antiviral treatment. The remainder of the patients would be regarded as gray-zone patients. These patients had been long-term patients who had been followed at the liver clinic of the institution, and a review of the records did not show any fluctuations of ALT levels. They also had no or minimal fibrosis as indicated by both Fibroscan and Fibrotest and normal baseline ALT, and therefore they would also not require treatment as suggested by current Western guidelines [4]. However, updated guidelines may consider the treatment of this group of gray-zone patients, as more and more data now suggest improved clinical outcomes in this subset of patients.

Several recent reports have shown that antiviral treatment in HBeAg-negative patients can achieve HBsAg clearance at a rate higher than that of spontaneous HBsAg clearance of approximately 1% per year [24]. The functional cure of HBV defined as undetectable HBsAg and HBV DNA is regarded as prognostically important as it is associated with better clinical outcomes compared to those who remain HBsAg positive with undetectable HBV DNA [25]. In a real-world study of 32 HBeAg-negative patients with very low levels of HBsAg (<20 IU/mL) treated with weekly injections of pegylated interferon (PEG IFN) 180 µg for a mean of 20 weeks, 93.8% (15/16) patients achieved HBsAg loss, and five of these patients (33% of responders) underwent HBsAg seroconversion when followed for a mean of 12 months [26], whereas none of the control subjects lost HBsAg (*p* < 0.0001). In another nonrandomized study of 144 inactive carriers, patients could opt for treatment versus observation. Those with HBV DNA < 20 IU/mL received weekly PEG IFN 180 µg/week alone, while those with HBV DNA ≥ 20 IU/mL received a combination of PEG IFN 180 µg/week plus adefovir 10 mg daily for a total duration of 96 weeks. HBsAg clearance and seroconversion at week 96 among those who received treatment was 44.7% and 38.3%, respectively, compared to only 2.4% and 0% in the observation group [27]. In another randomized controlled trial, patients with low HBV DNA (<20,000 IU/mL) received either PEG IFN plus a nucleos(t)ide analogue (NA) (n = 103) or no treatment (n = 48) for 48 weeks [28]. Four (4%) patients in the combination treatment group achieved HBsAg loss, whereas no HBsAg loss was observed in the control group (*p* = 0.377). Clearly, these disparate results are related to the fact that different subgroups of HBeAg-negative patients were studied, and different regimens of variable duration were used. In addition, predictors of HBsAg loss were not identified, which was likely a reflection of the low number of patients in each of these studies and the relatively homogeneous hepatitis B genotype distribution in this population.

In this study, we chose to use TDF, a nucleotide analogue, as monotherapy, in order to avoid the potential side effects of PEG IFN. TDF has demonstrated a favorable safety profile and potent antiviral activity without the development of antiviral resistance [29]. Although the use of TDF is associated with a small risk for renal impairment, the risk is greater in those over the age of 60 years, elevated bilirubin levels and pre-existing diabetes mellitus and hypertension [30] Clinically significant loss in bone mineral density or increased fractures were not observed in patients who received TDF continuously for 3 years or more [31,32,33]. More recently, tenofovir alafenamide (TAF) has been approved for use in patients with CHB infection, and it is recommended as a first-line therapy along with TDF and entecavir in most treatment guidelines. Long-term studies have confirmed that TAF has superior renal and bone safety compared to TDF [34]. However, TAF was not approved for use when this study was conducted. Therefore, we chose a treatment period of 3 years to minimize the potential side effects of TDF. The relative younger age of the study subjects, normal baseline serum bilirubin, normal baseline renal function and serum creatinine, and absence of metabolic comorbidity in most subjects tended to minimize the risk of renal dysfunction and accelerated bone loss.

Our data demonstrate that TDF induced HBV functional cure in 5 of 47 (11%) subjects who completed the study, 4 (9%) of whom went onto HBsAg seroconversion. It is worthwhile to note that all the cases of functional cure occurred in patients who were inactive carriers with a pretreatment HBsAg < 2000 IU/mL, thus providing a functional cure rate of 17.2% and a seroconversion rate of 13.8% amongst inactive carriers. This is significantly higher than the 1% per year spontaneous HBsAg loss rate amongst untreated patients [24]. Our findings are also in line with the published literature, which reported a higher incidence of HBsAg loss with treatment in patients who had lower baseline HBsAg levels [35]. It is interesting to note that at the end of the 6-month follow-up period, significantly more inactive carriers remained HBV DNA negative. It is possible that higher rates of HBsAg loss and seroconversion may be seen with a longer duration of off-treatment follow-up. This may be related to the fact that subjects in this study had low levels of fibrosis as indicated by their fibrosis scores. Incidentally, even though Fibrotest scores numerically increased during the study, the increase was small and clinically insignificant, as they all represented low level of fibrosis. 

There were no cases of hepatitis flares or clinical relapse after the withdrawal of TDF among our subjects. This is in contrast to other studies of treatment discontinuation in patients with HBeAg-negative CHB. In a small randomized study of stopping TDF vs. continuing therapy in mainly Asian patients, biochemical or virologic relapse was observed in 33% patients, and almost 40% required NA retreatment [36]. In that particular study, only one (2%) patient underwent HBsAg loss while off TDF. By contrast, in other studies of mainly Caucasian patients in Europe, HBsAg loss was reported in up to 20% off treatment [37]. This difference in rates of functional cure between these studies and our data can be explained by different HBV genotypes among different populations, heterogeneity in pretreatment HBeAg status, variability in treatment duration, the presence of metabolic dysfunction-associated liver disease and lower HBsAg levels at baseline and at the time of treatment withdrawal.

This study has several limitations. This was an open-label pilot study of consecutive subjects and not a randomized controlled trial, which could have introduced some bias. The majority of subjects were Asian, and the results may not be generalizable to other patients due to the known racial differences in HBV genotype. We decided empirically to treat our subjects for a period of 3 years to minimize the potential side effects of TDF, although the optimal period of NA treatment for HBeAg-negative patients with low HBV DNA is unknown. In addition, the duration of follow-up off TDF was relatively short at only 6 months. Despite this, we were able to show that a relatively short duration (3 years) of TDF was able to induce functional cure in 17% subjects who were in the inactive carrier state. 

In conclusion, TDF treatment for 3 years in patients with HBeAg-negative chronic infection currently not recommended for antiviral therapy resulted in significantly higher than expected rates of functional cure compared to untreated HBV subjects, particularly amongst inactive carriers with low baseline HBsAg < 2000 IU/mL and low HBV DNA < 2000 IU/mL. Our results suggest potential clinical benefits and improved prognosis as a result of TDF treatment in patients with HBeAg-negative chronic infection who achieve HBsAg loss. A similar approach to treat patients who are inactive carriers has been considered [21], and there have been suggestions to treat all patients who are HBsAg positive in order to achieve eventual CHB eradication [38]. Although this potential expanded indication for TDF appears promising, our results need to be confirmed in larger randomized controlled studies of longer duration of NA therapy. Such studies will characterize the subgroup of patients who are most likely to achieve HBV functional cure on NA therapy of finite duration.

## Figures and Tables

**Table 1:** Patient laboratory parameters at various time points during the study.

	Baseline	M6	M12	M18	M24	M30	M36	M42	*p* Value
Hemoglobin (g/L)	142 ± 14	143 ± 14	144 ± 16	144 ± 15	142 ± 15	141 ± 15	143 ± 15	143 ± 14	0.99
WBC (×10^9^/L)	5.5 ± 1.2	5.4 ± 1.2	5.9 ± 1.8	5.5 ± 1.3	5.7 ± 2.5	5.4 ± 1.3	5.3 ± 1.4	5.2 ± 1.2	0.48
INR	0.98 ± 0.06	0.98 ± 0.06	0.97 ± 0.07	0.97 ± 0.06	0.99 ± 0.06	0.98 ± 0.07	1.00 ± 0.06	0.99 ± 0.07	0.50
Serum sodium (mmol/L)	139 ± 2	139 ± 2	139 ± 2	139 ± 2	139 ± 2	139 ± 2	140 ± 2	139 ± 2	0.67
Serum creatinine (µmol/L)	70 ± 11	74 ± 12	73 ± 12	73 ± 11	73 ± 11	75 ± 14	74 ± 13	73 ± 11	0.73
eGFR (ml/min/1.73 m^2^)	95 ± 12	93 ± 13	94 ± 12	94 ± 13	92 ± 14	91 ± 14	91 ± 14	93 ± 14	0.75
AST (IU/L)	21 ± 5	26 ± 8	24 ± 6	25 ± 6	25 ± 6	25 ± 6	24 ± 5	22 ± 5	0.004
ALT (IU/L)	21 ± 8	26 ± 11	24 ± 10	26 ± 10	24 ± 9	26 ± 12	25 ± 11	21 ± 9	0.022
ALP (IU/L)	64 ± 17	75 ± 20	77 ± 20	73 ± 20	75 ± 21	72 ± 19	74 ± 20	67 ± 18	0.016
γGT (IU/L)	21 ± 12	23 ± 13	24 ± 16	23 ± 12	24 ± 19	23 ± 13	24 ± 15	24 ± 12	0.98
Bilirubin (µmol/L)	11 ± 6	11 ± 4	12 ± 6	11 ± 6	12 ± 7	11 ± 7	12 ± 7	12 ± 7	0.91
Albumin (g/L)	44 ± 2	44 ± 2	44 ± 3	43 ± 2	43 ± 3	43 ± 2	44 ± 3	44 ± 3	0.32
Protein (g/L)	76 ± 4	76 ± 4	77 ± 5	76 ± 4	77 ± 4	76 ± 4	76 ± 4	77 ± 4	0.59
HBV DNA (IU/mL) *	1220 (189:4980)	0 (0:9)	0 (0:9)	0 (0:9)	0(0:0)	0 (0:9)	0(0:0)	84 (9:430)	<0.001
HBsAg (IU/mL)	1074 (83:2948)	803 (68:3066)	847 (71:2681)	962 (73:2761)	968 (85:3143)	1029 (78:3374)	891 (62:3200)	563 (7:3453)	1.00
Fibroscan score	5.2 ± 1.6	-	4.7 ± 1.4	-	4.7 ± 1.1	-	4.7 ± 1.3	-	0.26
Fibrotest	0.23 ± 0.15		0.29 ± 0.15		0.30 ± 0.16		0.31 ± 0.16	0.27 ± 0.14	0.048

ALT: alanine aminotransferase; AST: aspartate aminotransferase; ALP: alkaline phosphatase; eGFR: estimated glomerular filtration rate; γGT: gamma glutamyl transferase; HBsAg: quantitative hepatitis B surface antigen; HBV DNA: hepatitis B DNA; INR: International normalized ratio; WBC: white cell count. All values are expressed as mean ± standard deviation. * Values are expressed as median and interquartile ranges.

## Data Availability

Data is unavailable due to privacy and ethical restrictions.

## Supplementary Materials

The following supporting information can be downloaded at https://www.mdpi.com/article/10.3390/jcm13071864/s1.

## Abbreviations

ALT	alanine aminotransferase
CHB	chronic hepatitis B
DNA	deoxyribonucleic acid
HBeAg	hepatitis B e antigen
HBeAb	hepatitis B e antibody
HBsAg	hepatitis B surface antigen
HBsAb	hepatitis B surface antibody
HBV	hepatitis B virus
HCC	hepatocellular carcinoma
IQR	interquartile range
NA	nucleos(t)ide analogue
PEG IFN	pegylated interferon
TAF	tenofovir alafenamide
TDF	tenofovir disoproxil fumarate

## References

[1] WHO (2021). Hepatitis B. https://www.who.int/news-room/fact-sheets/detail/hepatitis-b.

[2] https://www150.statcan.gc.ca/n1/daily-quotidien/221026/dq221026a-eng.htm.

[3] https://www.canada.ca/en/public-health/services/publications/diseases-conditions/report-hepatitis-b-c-canada-2018.html.

[4] Jeng W.J., Papatheodoridis G.V., Lok A.S.F. (2023). Hepatitis B. Lancet.

[5] Terrault N.A., Bzowej N.H., Chang K.M., Hwang J.P., Jonas M.M., Murad M.H., American Association for the Study of Liver Diseases (2016). AASLD guidelines for treatment of chronic hepatitis B. Hepatology.

[6] Lampertico P., Agarwal K., Berg T., Buti M., Janssen H.L., Papatheodoridis G., Zoulim F., Tacke F. (2017). EASL 2017 Clinical Practice Guidelines on the management of hepatitis B virus infection. J. Hepatol..

[7] Sarin S., Kumar M., Lau G.K., Abbas Z., Chan H.L.Y., Chen C.J., Chen D.S., Chen H.L., Chen P.J., Chien R.N. (2016). Asian-Pacific clinical practice guidelines on the management of hepatitis B: A 2015 update. Hepatol. Int..

[8] Abdo A.A., Bzeizi K.I., Babatin M.A., AlSohaibani F., AlMana H., Alsaad K.O., AlGhamdi H., Al-Hamoudi W., AlSwat K., AlFaleh F.Z. (2014). Predictors of significant fibrosis in chronic hepatitis B patients with low viremia. J. Clin. Gastroenterol..

[9] Koffas A., Kumar M., Gill U.S., Jindal A., Kennedy P.T.F., Sarin S.K. (2021). Chronic hepatitis B: The demise of the ‘inactive carrier’ phase. Hepatol. Int..

[10] Kan Q., Wen J., Xue R. (2015). Discrimination against people with hepatitis B in China. Lancet.

[11] Yang T., Wu M.C. (2011). Discrimination against hepatitis B carriers in China. Lancet.

[12] Choi H.S.J., Tonthat A., Janssen H.L.A., Terrault N.A. (2022). Aiming for functional cure with established and novel therapies for chronic hepatitis B. Hepatol. Commun..

[13] Terrault N.A., Lok A.S., McMahon B.J., Chang K.M., Hwang J.P., Jonas M.M., Brown R.S., Bzowej N.H., Wong J.B. (2018). Update on prevention, diagnosis, and treatment of chronic hepatitis B: AASLD 2018 hepatitis B guidance. Hepatology.

[14] Tai D.I., Lin S.M., Sheen I.S., Chu C.M., Lin D.Y., Liaw Y.F. (2009). Long-term outcome of hepatitis B e antigen-negative hepatitis B surface antigen carriers in relation to changes of alanine aminotransferase levels over time. Hepatology.

[15] Hadziyannis S.J., Vassilopoulos D. (2001). Hepatitis B e antigen-negative chronic hepatitis B. Hepatology.

[16] Chen Y.C., Huang S.F., Chu C.M., Liaw Y.F. (2012). Serial HBV DNA levels in patients with persistently normal transaminase over 10 years following spontaneous HBeAg seroconversion. J. Viral Hepat..

[17] Kumar M., Sarin S.K., Hissar S., Pande C., Sakhuja P., Sharma B.C., Chauhan R., Bose S. (2008). Virological and histological features of chronic hepatitis b virus infected asymptomatic patients with persistently normal ALT. Gastroenterology.

[18] Chu C.M., Liaw Y.F. (2007). Spontaneous relapse of hepatitis in inactive HBsAg carriers. Hepatol. Int..

[19] Chu C.M., Liaw Y.F. (2007). Predictive factors for reactivation of hepatitis B following hepatitis B e antigen seroconversion in chronic hepatitis B. Gastroenterology.

[20] Coffin C.S., Fung S.K., Alvarez F., Cooper C.L., Doucette K.E., Fournier C., Kelly E., Ko H.H., Ma M.M., Martin S.R. (2018). Management of Hepatitis B Virus Infection: 2018 Guidelines from the Canadian Association for the Study of the Liver and Association of Medical Microbiology and Infectious Disease Canada. Can. Liver J..

[21] You H., Wang F.S., Li T., Xu X., Sun Y., Nan Y., Wang G., Hou J., Duan Z., Wei L. (2023). Guidelines for the prevention and treatment of chronic hepatitis B (version 2022). J. Clin. Transl. Hepatol..

[22] Bonacci M., Lens S., Mariño Z., Londoño M.C., Rodríguez-Tajes S., Mas A., García-López M., Pérez-del-Pulgar S., Sánchez-Tapias J.M., Forns X. (2018). Anti-viral therapy can be delayed or avoided in a significant proportion of HBeAg-negative Caucasian patients in the Grey Zone. Aliment. Pharmacol. Ther..

[23] Teng W., Chang T.T., Yang H.I., Peng C.Y., Su C.W., Su T.H., Hu T.H., Yu M.L., Yang H.C., Wu J.C. (2021). Risk scores to predict HCC and the benefits of antiviral therapy for CHB patients in gray zone of treatment guidelines. Hepatol. Int..

[24] Chu C.M., Liaw Y.F. (2007). HBsAg seroclearance in asymptomatic carriers of high endemic areas: Appreciably high rates during a long-term follow-up. Hepatology.

[25] Anderson R.T., Choi H.S., Lenz O., Peters M.G., Janssen H.L., Mishra P., Donaldson E., Westman G., Buchholz S., Miller V. (2021). Association between seroclearance of hepatitis B surface antigen and long-term clinical outcomes of patients with chronic hepatitis B virus infection: Systematic review and meta-analysis. Clin. Gastroenterol. Hepatol..

[26] Zeng Q.L., Yu Z.J., Shang J., Xu G.H., Sun C.Y., Liu N., Li C.X., Lv J., Liu Y.M., Liang H.X. (2020). Short-term peginterferon-Induced high functional cure rate in inactive chronic hepatitis B virus carriers with low surface antigen levels. Open Forum Infect. Dis..

[27] Cao Z., Liu Y., Ma L., Lu J., Jin Y., Ren S., He Z., Shen C., Chen X. (2017). A potent hepatitis B surface antigen response in subjects with inactive hepatitis B surface antigen carrier treated with pegylated-interferon alpha. Hepatology.

[28] de Niet A., Jansen L., Stelma F., Willemse S.B., Kuiken S.D., Weijer S., van Nieuwkerk C.M., Zaaijer H.L., Molenkamp R., Takkenberg R.B. (2017). Peg-interferon plus nucleotide analogue treatment versus no treatment in patients with chronic hepatitis B with a low viral load: A randomised controlled, open-label trial. Lancet Gastroenterol. Hepatol..

[29] Marcellin P., Wong D.K., Sievert W., Buggisch P., Petersen J., Flisiak R., Manns M., Kaita K., Krastev Z., Lee S.S. (2019). Ten-year efficacy and safety of tenofovir disoproxil fumarate treatment for chronic hepatitis B virus infection. Liver Int..

[30] Jung W.J., Jang J.Y., Park W.Y., Jeong S.W., Lee H.J., Park S.J., Lee S.H., Kim S.G., Cha S.W., Kim Y.S. (2018). Effect of tenofovir on renal function in patients with chronic hepatitis B. Medicine.

[31] Wang H.M., Hung C.H., Lee C.M., Lu S.N., Wang J.H., Yen Y.H., Kee K.M., Chang K.C., Tseng P.L., Hu T.H. (2016). Three-year efficacy and safety of tenofovir in nucleos(t)ide analog-naïve and nucleos(t)ide analog-experienced chronic hepatitis B patients. J. Gastroenterol. Hepatol..

[32] Fung S., Kwan P., Fabri M., Horban A., Pelemis M., Hann H.W., Gurel S., Caruntu F.A., Flaherty J.F., Massetto B. (2017). Tenofovir disoproxil fumarate (TDF) vs. emtricitabine (FTC)/TDF in lamivudine resistant hepatitis B: A 5-year randomised study. J. Hepatol..

[33] Wei M.T., Le A.K., Chang M.S., Hsu H., Nguyen P., Zhang J.Q., Wong C., Wong C., Cheung R., Nguyen M.H. (2019). Antiviral therapy and the development of osteopenia/osteoporosis among Asians with chronic hepatitis B. J. Med. Virol..

[34] Chan H.L., Buti M., Lim Y.S., Agarwal K., Marcellin P., Brunetto M., Chuang W.L., Janssen H.L., Fung S., Izumi N. (2023). Long-term treatment with tenofovir alafenamide for chronic hepatitis B results in high rates of viral suppression and favorable renal and bone safety. Am. J. Gastroenterol..

[35] Sonneveld M.J., Chiu S.M., Park J.Y., Brakenhoff S.M., Kaewdech A., Seto W.K., Tanaka Y., Carey I., Papatheodoridi M., van Bömmel F. (2022). Probability of HBsAg loss after nucleo(s)tide analogue withdrawal depends on HBV genotype and viral antigen levels. J. Hepatol..

[36] Liem K.S., Fung S., Wong D.K., Yim C., Noureldin S., Chen J., Feld J.J., Hansen B.E., Janssen H.L. (2019). Limited sustained response after stopping nucleos(t)ide analogues in patients with chronic hepatitis B: Results from a randomised controlled trial (Toronto STOP study). Gut.

[37] Berg T., Simon K.G., Mauss S., Schott E., Heyne R., Klass D.M., Eisenbach C., Welzel T.M., Zachoval R., Felten G. (2017). Long-term response after stopping tenofovir disoproxil fumarate in non-cirrhotic HBeAg-negative patients—Finite study. J. Hepatol..

[38] Roma K., Dossaji Z., Haque L., Laeeq T., Gish R.G., Brosgart C. (2023). Test all for hepatitis B virus: Link to care and treatment if quantitative DNA positive, vaccinate if susceptible. Clin. Liver Dis..

