# Supplementary figures and images for: The Use of Tenofovir Disoproxil Fumarate in the Management of eAg-Negative Chronic Hepatitis B Infection
Source: J Clin Med. 2024 Mar 24;13(7):1864. doi: 10.3390/jcm13071864 (PMC11012673; doi:10.3390/jcm13071864)

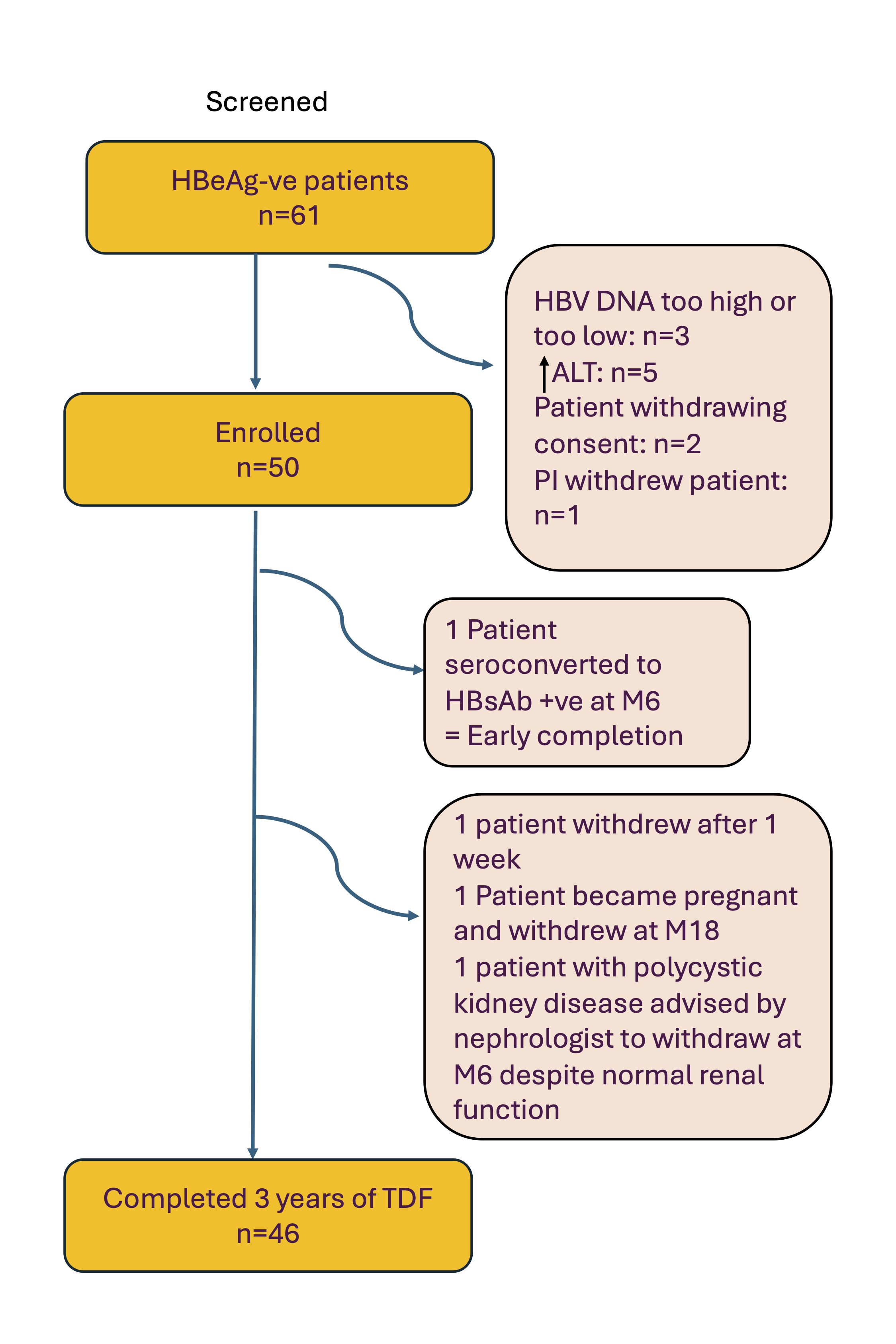

Supplement: Supplementary file 1 [file jcm-13-01864-s001.zip › jcm-2892192-supplementary.tiff]
